# Early-life respiratory syncytial virus lower respiratory tract infection in a South African birth cohort: epidemiology and effect on lung health

**DOI:** 10.1016/S2214-109X(20)30251-5

**Published:** 2020-09-21

**Authors:** Heather J Zar, Polite Nduru, Jacob A M Stadler, Diane Gray, Whitney Barnett, Maia Lesosky, Landon Myer, Mark P Nicol

**Affiliations:** a Department of Paediatrics and Child Heath, Red Cross War Memorial Children's Hospital and South African Medical Research Council Unit on Child and Adolescent Health, University of Cape Town, Cape Town, South Africa; b Division of Epidemiology and Biostatistics, School of Public Health and Family Medicine, University of Cape Town, Cape Town, South Africa; c Division of Medical Microbiology, University of Cape Town, Cape Town, South Africa; d Division of Infection and Immunity, Department of Biomedical Sciences, University of Western Australia, Perth, WA, Australia

## Abstract

**Background:**

Respiratory syncytial virus (RSV) is a major cause of lower respiratory tract infection (LRTI) in children. Early-life RSV LRTI might affect long-term health but there are few data from low-income and middle-income countries. We investigated the epidemiology and effect of early-life RSV LRTI on lung health in a South African birth cohort.

**Methods:**

We conducted the Drakenstein Child Health Study (DCHS), an ongoing birth cohort longitudinal study in the Western Cape province, South Africa. We enrolled pregnant women aged 18 years or older during their second trimester of pregnancy at two public health clinics. We followed up study children from birth to 2 years. The primary outcome of the study was LRTI and RSV LRTI. LRTI and wheezing episodes were identified through active surveillance; respiratory samples were tested for RSV and other pathogens. Wheezing was longitudinally identified by caregiver report and ascertainment at health facilities. Lung function was measured from 6 weeks to 2 years. We analysed the associations between RSV LRTI and subsequent LRTI, wheezing, and lung function using generalised estimating equations and mixed-effects linear regression.

**Findings:**

We enrolled 1137 mothers between March 5, 2012, and March 31, 2015. Among their 1143 infants, accruing 2093 child-years of follow-up, there were 851 cases of LRTI (incidence 0·41 episodes per child-year, 95% CI 0·38–0·43). Admission to hospital owing to LRTI occurred in 169 (20%) cases (incidence 0·08 episodes per child-year, 0·07–0·09), with a case-fatality ratio of 0·5%. RSV was detected in 164 (21%) of 785 LRTI events with a specimen available for qPCR, an incidence of 0·08 episodes per child-year (0·07–0·09); highest at age 0–6 months (0·15 episodes per child-year, 0·12–0·19). Children with a first RSV LRTI were three times as likely to develop recurrent LRTI compared with those with non-RSV LRTI (0·32 [0·22–0·48] *vs* 0·10 [0·07– 0·16] episodes per child-year; p<0·0001), particularly following hospitalised RSV LRTI. RSV LRTI and hospitalisation for all-cause LRTI were independently associated with recurrent wheezing (adjusted incident rate ratio 1·41, 95% CI 1·25–1·59, for RSV LRTI and 1·48, 1·30–1·68, for hospitalisation). LRTI or recurrent LRTI was associated with impaired lung function, but a similar outcome was observed following RSV LRTI or non-RSV LRTI. All-cause LRTI was associated with an average 3% higher respiratory rate (95% CI 0·01–0·06; p=0·013) and lower compliance (–0·1, −0·18 to 0·02) at 2 years compared with no LRTI. Recurrent LRTI was associated with further increased respiratory rate (0·01, 0·001–0·02), resistance (0·77 hPa s L^−1^, 0·07–1·47), and lower compliance (–0·6 mL hPa^−1^, −0·09 to −0·02) with each additional event.

**Interpretation:**

RSV LRTI was common in young infants and associated with recurrent LRTI, particularly after hospitalised RSV. Hospitalisation for all-cause LRTI, especially for RSV-LRTI, was associated with recurrent wheezing. Impairments in lung function followed LRTI or recurrent episodes, but were not specific to RSV. New preventive strategies for RSV might have an effect on long-term lung health.

**Funding:**

Bill & Melinda Gates Foundation; South African Medical Research Council; National Research Foundation South Africa; National Institutes of Health, Human Heredity and Health in Africa; Wellcome Trust.

## Introduction

Respiratory syncytial virus (RSV) is a leading cause of childhood lower respiratory tract infection (LRTI) and mortality globally, particularly in low-income and middle-income countries (LMICs).^1^, ^2^ With reductions in bacterial pneumonia following pneumococcal conjugate vaccine (PCV) and *Haemophilus influenzae* type b (Hib) vaccine, RSV is associated with an increasing aetiological fraction of LRTI.^2^ Infants have the highest risk of disease and of mortality,^3^ but the burden of disease beyond infancy has not been well studied in longitudinal cohort studies, especially in LMICs.

As countries achieve reductions in under-5 mortality, the potential effect of early-life RSV LRTI on long-term morbidity is increasingly important. RSV LRTI has been associated with an increased risk of recurrent wheezing or asthma in childhood, but whether this is causal, a marker of underlying susceptibility, or both is unclear.^4^ Systematic reviews and meta-analyses report an association between early-life RSV LRTI and recurrent wheezing or asthma, particularly for RSV hospitalisation.^4^, ^5^, ^6^ However, in some studies the association was transient or reduced with age. Two randomised controlled trials of monoclonal antibodies to prevent RSV LRTI in infants did not find long-term differences in wheezing or asthma prevalence. Dutch parents reported fewer wheezing days in the first year amongst premature infants who received palivizumab^7^ but by age 6 years no difference existed in doctor-diagnosed asthma or in lung function between palivizumab and placebo arms.^8^ A second study of motavizumab in full-term Native American infants, followed up to 3 years,^9^ reported no difference in medically attended wheezing between those who received monoclonal antibody or placebo.

**Research in context**

**Evidence before this study**

We searched PubMed for articles published in English up until Sept 15, 2019, using the search terms “child” and “lower respiratory tract infection (LRTI)” and “RSV” and “wheezing” or “lung function”. We identified four systematic reviews or meta-analyses reporting an association with respiratory syncytial virus (RSV) LRTI and subsequent asthma or wheezing. However, most studies included were from high-income countries and some reported only a transient association. We also identified four randomised controlled studies of monoclonal antibodies given to infants for prevention of RSV, which reported conflicting results for subsequent wheezing without lung function measurements. We identified only one study reporting on recurrent LRTI following RSV. We identified very few studies with lung function measurements before and after LRTI, with few, small studies from high-income countries reporting different findings on the association between RSV LRTI and subsequent lung function impairment.

**Added value of this study**

In a unique African birth cohort, the Drakenstein Child Health study, we longitudinally investigated LRTI, RSV LRTI, lung function, and subsequent development of recurrent LRTI or wheezing from birth to age 2 years. We found that the incidence of LRTI was high, despite good nutrition, that very few children were HIV-infected (two of 1143), and that excellent immunisation coverage including 13-valent pneumococcal conjugate vaccine was in place. RSV was a major cause of LRTI, occurring in approximately 20% of episodes. The incidence and severity of RSV LRTI was highest in infants under 6 months, but RSV continued to be associated with approximately a fifth of LRTI up to age 2 years. A first episode of RSV LRTI, especially if severe, was associated with an increased risk of subsequent non-RSV recurrent LRTI. RSV was also associated with recurrent wheezing in early childhood, with the highest proportion following RSV LRTI hospitalisation. However, hospitalisation with non-RSV LRTI was also associated with an increased risk of recurrent wheezing. LRTI, particularly recurrent illness, was also associated with impairments in lung function at 2 years (lower compliance, higher respiratory rate, and respiratory system resistance), but this was not specific to RSV.

**Implications of all the available evidence**

Early-life LRTI remains an important public health problem and is associated with lung function impairment in childhood. RSV LRTI, especially severe disease, is associated with subsequent morbidity including recurrent non-RSV LRTI and wheezing. Severe all-cause LRTI is also associated with recurrent wheezing. New vaccines and novel strategies to prevent LRTI are needed; novel strategies to prevent RSV might reduce RSV LRTI, but might also have a larger public health impact on the incidence of subsequent LRTI and on chronic lung disease.

The effect of early-life RSV LRTI on recurrent wheezing or asthma is a key concern, given the large burden of this chronic disease. Asthma, now regarded as a spectrum of chronic airway diseases manifesting as recurrent wheezing,^10^ is the commonest chronic non-communicable disease in children, including in LMICs.^11^ Asthma prevalence is rising in LMICs, where disease is more severe than in high-income settings.^11^ With several new RSV preventive interventions in development or undergoing clinical trials, understanding the association of RSV LRTI with recurrent wheezing is relevant to assessing additional potential effect of these interventions.^12^

In 2016, we reported a high incidence of LRTI in infants enrolled in the Drakenstein Child Health study (DCHS), a South African birth cohort study.^13^ RSV was the commonest viral pathogen associated with LRTI. The aim of this study was to longitudinally investigate the epidemiology of RSV LRTI from birth to age 2 years and the effect of RSV LRTI on subsequent recurrent lung health including wheezing and lung function.

## Methods

### Study design and participants

The DCHS, located in a peri-urban area in South Africa, enrolled pregnant women from March, 2012, to March, 2015, during their second trimester of pregnancy at two public health clinics.^14^ Inclusion criteria were age 18 years or older, 20–28 weeks' gestation, and resident in the area. Study visits were synchronised with health-care and immunisation visits (diphtheria, tetanus, acellular pertussis, Hib, and inactivated polio vaccine at 6, 10, 14 weeks, and 18 months, measles vaccine at 9 and 18 months, and 13-valent PCV [PCV13] at 6 weeks, 14 weeks, and 9 months), with additional study visits at 6, 12, and 24 months. Mother–infant pairs could also participate in intensive one follow-up visit every 2 weeks during the first year.^14^

We followed up children from birth until age 2 years for LRTI or wheezing using active surveillance systems, as described.^15^ Continuous surveillance was implemented at local clinics and at Paarl hospital. WHO criteria were used to define pneumonia or LRTI, as previously described.^13^ Children were followed up throughout LRTI hospital admission, or following an ambulatory episode. Recurrent LRTI was defined as two or more episodes. Episodes of wheezing were reported by a caregiver using questions adapted from the International Study of Asthma and Allergies in Childhood or were diagnosed on auscultation by trained study staff at a study visit or during an intercurrent illness.^16^

Longitudinal measurement of risk factors for LRTI or wheezing including nutrition, home environment, vaccinations, smoke exposure, and maternal factors was done at study visits and during illness. Maternal smoking or passive smoke exposure was self-reported. A composite locally validated measure from the South African Stress and Health Study^17^ of socioeconomic status was used encompassing current employment, education, household income, and an asset index.

The study was approved by the Faculty of Health Sciences Research Ethics Committee, University of Cape Town and Western Cape Provincial Research committee. Mothers gave written informed consent at enrolment and re-consented annually.

### Procedures

At each LRTI or wheezing episode, a nasopharyngeal swab (FLOQSwabs, Copan Diagnostics, Murrieta, CA, USA) was obtained. Nucleic acid was extracted using mechanical lysis on a Tissuelyzer LT (Qiagen, Hilden, Germany) followed by extraction with the QIAsymphony Virus/Bacteria mini kit (Qiagen). Quantitative multiplex real-time PCR (qPCR) was done using FTDResp33 (Fast Track Diagnostics, Esch-sur-Alzette, Luxembourg), identifying up to 33 organisms including RSV. RSV LRTI was defined as any episode of LRTI that was positive for RSV on qPCR.

Comprehensive, validated^18^ lung function measurements were done using the multiple breath washout technique (measuring lung volume, functional residual capacity and the lung clearance index [LCI]); tidal breathing (measuring respiratory rate, tidal volume, and flow rates); respiratory impedance, resistance and compliance, and exhaled nitric oxide in 6-week-old unsedated sleeping infants and at 1 and 2 years, as described.^18^ Lung function was done when infants and children were healthy, and after 4 weeks of a LRTI.

### Outcomes and analysis

The primary outcome of the study was LRTI and RSV LRTI. Data were analysed using Stata version 14.1 and R. Socioeconomic status was evaluated in quartiles. Wilcoxon rank-sum test and χ^2^ or Fisher's exact were used for crude comparison, as appropriate. Child follow-up period was divided into intervals of 2 weeks that were used to calculate person-time at risk, with a not at risk (excluded person-time) period of 2 weeks after an LRTI or 4 weeks (28 days) after a wheezing episode. LRTI incidence was reported as episodes per child-year with 95% CI. Episodes of LRTI occurring immediately after birth or before discharge after delivery were regarded as congenital events and were excluded from this analysis.

To examine independent predictors of LRTI we used negative binomial regression with a log link during each follow-up interval; generalised estimating equations were used to account for intraindividual clustering of intervals. We used these models to compare RSV LRTI versus non-RSV LRTI, or hospitalised LRTI versus ambulatory LRTI. Separate models included all individuals in the cohort and individuals with any episode of LRTI versus no LRTI. Multivariable models were adjusted for an a-priori set of confounding factors, identified through expert consultation with investigators and construction of a directed acyclic graph to identify the minimum covariates for models. A-priori confounders included were: sex, socioeconomic status quartile, season at birth, other children in the household, HIV exposure, maternal smoking, prematurity, low birthweight, and age at mid-interval or age at LRTI if an episode occurred in the interval. Multivariable modelling of risk factors associated with LRTI was done for any LRTI versus no LRTI (model A); RSV LRTI versus no LRTI (model B); and RSV LRTI versus non-RSV LRTI (model C).

Recurrent wheezing was defined a-priori as two or more observations of wheezing after the LRTI event in individuals experiencing LRTI and two or more observations of wheezing in those who did not experience an LRTI. Similar to LRTI, wheezing models were based on generalised estimating equations with a negative binomial family and link function, adjusting for similar covariates with age included at time of reported or assessed wheeze or mid-interval of no wheeze recorded. For children with recurrent wheezing, time to first wheezing event after LRTI was used as the time to event for product-limit analyses. Additional analyses for three or more observations of wheezing was also done. For the Kaplan Meier estimates, survival proportions (cumulative incidences from survival analyses) were used. Child follow-up period was calculated as described.

Longitudinal changes in lung function measures from 6 weeks to 2 years were analysed using mixed-effects linear models, with random effects for lung function outcomes, thus accounting for baseline lung function in models. These were fitted using an a-priori set of confounding factors of body-mass index for age *Z* score, ethnicity, sex, socioeconomic status quartile, age at time of lung function test, and gestational age at birth.

### Role of the funding source

Study sponsors had no role in the study design; collection, analysis, and interpretation of the data; writing of the report; or the decision to submit for publication. All authors had full access to all the data and share final responsibility for the decision to submit for publication.

## Results

**Figure 1:**
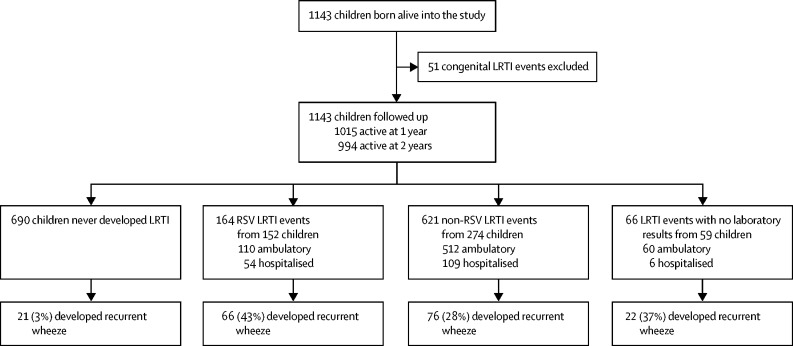
Study flow diagram

Among 1137 mothers (median age 26 [IQR 22–31] years) enrolled from March 5, 2012, to March 31, 2015, there were 1143 livebirths (four sets of twins, one set of triplets) providing 2093 child-years of follow-up (table 1). Of 1143 births, cohort retention was 89% (1015) at 1 year and 87% (994) at 2 years (figure 1). Most mothers (879 [77%]) chose to participate in intensive follow up of one visit every 2 weeks. Most disenrollment (125 [11%]) was due to relocation (29 [2·5%]) or inability to contact the mother (33 [2·9%]), disenrollment followed at least three unsuccessful attempts (phone calls and home visits) by study staff to locate participants. Child mortality was 2% (24 of 1143), with six deaths attributable to pneumonia (pneumonia-specific mortality, 0·5%). Most households (87%) had a median monthly income under R5000 (US$333); 579 (51%) had a parent employed (table 1). 244 (22%) mothers were HIV-infected, of whom 236 (96·7%) were on antiretroviral therapy; only two (<1%) children were HIV-infected (table 1). Smoking during pregnancy occurred in 27% of mothers and in 30% postnatally. Most mothers (1046 [92%] of 1137) initiated breastfeeding but duration of exclusive breastfeeding was short (out of 1068: 503 [47%] at 6 weeks and 279 [26%] at 3 months). Immunisation coverage was high with 100% coverage for week 6, 10, and 14 vaccines, 99% for 9 month, and 92% for 18 month vaccines (table 1). Only 2% (25 of 1130) of mothers reported having asthma or a previous diagnosis of asthma; family history of asthma or atopy was also low (53 [5%] of 987). Participants lost to follow-up were similar to those who completed follow-up, with similar rates of maternal HIV, employment, access to household electricity or running water, and gestational age and birthweight of infants.

**Table 1:** Characteristics of DCHS study population

		Overall (n=1137 mothers; n=1143 infants)*	**No LRTI**†**(n=688 mothers; n=690 infants)**	**LRTI**‡**(n=453 infants; 851 episodes)**§	**Hospitalised LRTI**‡**(n=130 infants; 169 episodes)**
**Maternal characteristics (n=1137)**					
Age (years)		26 (22–31)	25 (22–30)	26 (22–31)	26 (21–31)
Currently married or cohabiting		458/1137 (40%)	277/688 (40%)	181/449 (40%)	43/129 (33%)
Self-reported smoking antenatal		306/1134 (27%)	170/685 (25%)	136/449 (30%)	40/129 (31%)
Self-reported smoking postnatal		323/1061 (30%)	180/622 (29%)	143/439 (33%)	43/125 (34%)
HIV infection		244/1134 (22%)	127/686 (19%)	117/448 (26%)	33/129 (26%)
History of asthma or atopy		25/1130 (2%)	14/686 (2%)	11/444 (2%)	4/128 (3%)
Family history of asthma or atopy		53/987 (5%)	28/555 (5%)	25/432 (6%)	9/120 (8%)
**Infant characteristics (n=1143)**					
Lives in formal home or flat		617/1143 (54%)	378/688 (55%)	239/453 (53%)	69/130 (53%)
At least one parent employed		579/1143 (51%)	357/688 (52%)	222/453 (49%)	53/130 (41%)
Electricity in the home		1071/1139 (94%)	650/689 (94%)	421/450 (94%)	123/128 (96%)
Plumbing in the home		973/1140 (85%)	602/689 (87%)	371/451 (82%)	112/129 (87%)
Household income per month					
	<R1000 (US$67)	407/1114 (37%)	245/674 (36%)	162/440 (37%)	51/127 (40%)
	R1000–5000 (US$67–333)	553/1114 (50%)	331/674 (49%)	222/440 (50%)	56/127 (44%)
	R5000–10 000 (US$333–667)	128/1114 (11%)	81/674 (12%)	47/440 (11%)	16/127 (13%)
	>R10 000 (US$667)	26/1114 (2%)	17/674 (3%)	9/440 (2%)	4/127 (3%)
Crowding: individuals per house		4 (3–6)	4 (3–6)	4 (3–6)	5 (3–6)
Gestational age (weeks)		39 (38–40)	39 (38–40)	39 (37–40)	39 (37–40)
Premature (<37 weeks gestation)		194/1143 (17%)	111/688 (16%)	83/453 (18%)	32/130 (25%)
Gestational age (weeks) among premature births		35 (32–36)	35 (33–36)	34 (32–36)	34 (30–36)
Birthweight (kg)		3·08 (2·71–3·42)	3·12 (2·76–3·44)	3·02 (2·61–3·38)	2·97 (2·58–3·32)
HIV-infected		2/1143 (<1%)	1/690 (<1%)	1/453 (<1%)	1/130 (<1%)
HIV-exposed uninfected		246/1143 (22%)	127/690 (18%)	119/453 (26%)	32/130 (25%)
Initiated breastfeeding		1046/1137 (92%)	630/685 (92%)	417 (92%)	119/130 (92%)
Exclusive breastfeeding at 6 weeks		503/1068 (47%)	305/627 (49%)	198/441 (45%)	53/130 (42%)
Exclusive breastfeeding at 3 months		279/1068 (26%)	160/627 (26%)	119/441 (27%)	31/125 (25%)
Vaccination coverage					
	6 weeks	1039/1043 (>99%)	602/604 (100%)	437/439 (100%)	123/125 (98%)
	10 weeks	1024/1029 (>99%)	587/590 (99%)	437/439 (100%)	123/125 (98%)
	14 weeks	1013/1013 (100%)	578/578 (100%)	435/435 (100%)	123/123 (100%)
	9 months	964/974 (99%)	547/553 (99%)	417/421 (99%)	112/115 (97%)
	18 months	759/829 (92%)	442/478 (92%)	317/351 (90%)	95/103 (92%)
Age category at end of follow-up					
	0 to <1 year	128/1143 (11%)	115/688 (17%)	13/453 (3%)	7/130 (5%)
	1 to <2 years	21/1143 (2%)	17/688 (2%)	4/453 (1%)	1/130 (1%)
	≥2 years	994/1143 (87%)	558/688 (81%)	436/453 (96%)	122/130 (94%)
Follow-up (years)		2·05 (2·00–2·11)	2·02 (2·00–2·10)	2·06 (2·00–2·12)	2·06 (2·00–2·11)

Data are n/N (%); or median (IQR). LRTI=lower respiratory tract infection. DCHS=Drakenstein Child Health Study.

* Four sets of twins, one set of triplets.

† Includes 12 observations that ended before 14 days.

‡ Excludes congenital cases.

§ Includes the number of children ever developing LRTI in the first 2 years; however, some children had both RSV and non-RSV LRTI events.

There were 851 LRTI events (excluding 51 congenital episodes) in 453 children, giving a crude incidence of 0·41 episodes per child-year (95% CI 0·38–0·43, table 2). Incidence of LRTI was highest at 0–6 months of age (0·72, 0·65–0·79, episodes per child-year) and during winter (June–Aug; 0·52, 0·47–0·59, episodes per child-year). Among LRTI events, 169 (20%) of 851 required hospitalisation; the case-fatality ratio was 0·5%. Children hospitalised for LRTI were younger than those with ambulatory illness (median 6, IQR 3–13 months *vs* 8, 4–14 months; p<0·0001).

**Table 2:** Crude incidence rates as episodes per child-year for LRTI, hospitalised LRTI, RSV LRTI, and hospitalised RSV LRTI over the first 2 years of life in the DCHS

		**LRTI (n=851 episodes)**	**Hospitalised LRTI (n=169 episodes)**	**RSV LRTI (n=164 episodes)**	**Hospitalised RSV LRTI (n=54 episodes)**
All		0·41 (0·38–0·43)	0·08 (0·07–0·09)	0·08 (0·07–0·09)	0·03 (0·02–0·03)
Age group					
	0–6 months	0·72 (0·65–0·79)	0·17 (0·14–0·21)	0·15 (0·12–0·19)	0·07 (0·05–0·10)
	0–1 years	0·54 (0·50–0·59)	0·13 (0·11–0·15)	0·12 (0·10–0·14)	0·05 (0·03–0·06)
	1–2 years	0·28 (0·25–0·31)	0·04 (0·03–0·05)	0·04 (0·03–0·06)	0·01 (0·00–0·01)
Season					
	Autumn (Mar–May)	0·45 (0·40–0·51)	0·09 (0·06–0·11)	0·15 (0·12–0·19)	0·04 (0·03–0·06)
	Winter (June-Aug)	0·52 (0·47–0·59)	0·13 (0·10–0·16)	0·15 (0·12–0·19)	0·06 (0·04–0·08)
	Spring (Sept–Nov)	0·41 (0·36–0·47)	0·06 (0·04–0·09)	0·01 (0·00–0·02)	0·00 (0·00–0·002)
	Summer (Dec–Feb)	0·24 (0·20–0·28)	0·05 (0·03–0·07)	..	..
Severity					
	Hospitalised	0·08 (0·07–0·09)	..	0·03 (0·02–0·03)	..
	Ambulatory	0·33 (0·30–0·35)	..	0·05 (0·04–0·06)	..

Data are episodes per child-year (95% CI). LRTI=lower respiratory tract infection. RSV=respiratory syncytial virus. DCHS=Drakenstein Child Health Study.

Demographic characteristics of children who experienced an LRTI were similar to those of the overall cohort (table 1). Risk factors associated with LRTI included male sex (incident rate ratio [IRR] 1·48, 95% CI 1·22–1·79), lower socioeconomic status (low–moderate *vs* high 1·34, 1·02–1·77), HIV exposure (1·49, 1·20–1·86), low birthweight (1·48, 1·15–1·91), birth season (autumn *vs* summer 1·42, 1·11–1·81), and age (0·46, 0·40–0·52) (table 3, appendix p 1).

**Table 3:** Results of multivariable modelling of risk factors associated with any LRTI (model A) and RSV LRTI (models B and C) in children to 2 years in the DCHS

		**Model A: LRTI *vs* no LRTI**		**Model B: RSV LRTI *vs* no LRTI**		**Model C: RSV LRTI *vs* non-RSV LRTI**	
		Adjusted IRR (95% CI)	p value	Adjusted IRR (95% CI)	p value	Adjusted IRR (95% CI)	p value
Male sex (*vs* female)		1·48 (1·22–1·79)	<0·0001	1·16 (0·92–1·48)	0·21	0·77 (0·62–0·94)	0·01
Socioeconomic quartile (*vs* high)							
	Lowest	1·24 (0·92–1·65)	0·15	1·05 (0·72–1·54)	0·79	0·86 (0·62–1·18)	0·34
	Low–moderate	1·34 (1·02–1·77)	0·04	1·27 (0·91–1·77)	0·17	0·89 (0·66–1·19)	0·42
	Moderate–high	1·05 (0·79–1·39)	0·75	1·16 (0·81–1·65)	0·42	1·02 (0·76–1·36)	0·89
Presence of other children in the household		0·92 (0·66–1·28)	0·61	1·06 (0·71–1·60)	0·78	1·19 (0·84–1·69)	0·33
HIV exposed (*vs* HIV unexposed)		1·49 (1·20–1·86)	<0·0004	1·37 (1·04–1·81)	0·027	0·87 (0·69–1·11)	0·28
Maternal smoking after birth (self-reports; *vs* non-smoker)		1·09 (0·88–1·35)	0·43	1·33 (1·04–1·70)	0·025	1·38 (1·11–1·72)	0·0035
Preterm (<37 weeks' gestation)		1·20 (0·93–1·4)	0·17	1·06 (0·78–1·43)	0·72	0·92 (0·66–1·27)	0·60
Low birthweight (weight <2·5 kgs)		1·48 (1·15–1·91)	0·0023	1·31 (0·98–1·77)	0·070	0·92 (0·66–1·28)	0·62
Season of birth (*vs* summer)							
	Autumn (Mar–May)	1·42 (1·11–1·81)	0·0058	1·48 (1·11–1·97)	0·0071	1·13 (0·88–1·45)	0·35
	Winter (June–Aug)	0·93 (0·71–1·20)	0·57	0·77 (0·53–1·11)	0·16	0·86 (0·63–1·17)	0·35
	Spring (Sept–Nov)	0·97 (0·73–1·28)	0·83	0·58 (0·36–0·93)	0·024	0·65 (0·42–0·99)	0·046
Age at LRTI (or mid interval if no LRTI; years)		0·46 (0·40–0·52)	<0·001	1·00 (1·00–1·00)	<0·0001	0·79 (0·64–0·97)	0·025
Exclusive breastfeeding at 6 weeks		0·96 (0·79–1·17)	0·70	0·98 (0·77–1·25)	0·87	1·00 (0·80–1·24)	0·98

Results of generalised estimating equations expressed as incidence rate ratios (IRRs) and 95% CI. Models include all variables shown. LRTI=lower respiratory tract infection. RSV=respiratory syncytial virus. DCHS=Drakenstein Child Health Study.

Among 851 LRTI episodes, 66 (8%) did not have a specimen collected for qPCR, yielding 1143 children and 785 LRTI events for analysis (figure 1). RSV was detected in 164 (21%) episodes. Incidence of RSV LRTI was 0·08 episodes per child-year (95% CI 0·07–0·09) with age-specific incidence highest for infants 0–6 months (0·15 episodes per child-year, 0·12–0·19; table 2). Only five (1%) of 785 cases had no organism detected; a median of 6 (IQR 5–8) organisms occurred.

RSV was more common in hospitalised (33%, 54 of 163) than ambulatory LRTI (18%, 110 of 622; p<0·0001). Among 164 RSV LRTI episodes, 54 (33%) required hospitalisation and most (121 [74%]) occurred in infants (appendix p 2). Risk factors for RSV LRTI included maternal smoking, maternal HIV, season of birth, and child age (table 3). Children with RSV LRTI (median age 198 days, IQR 92–371) were younger than those with non-RSV LRTI (213 days, 112–437; p=0·074). RSV LRTI was commonest in autumn and winter (0·15 episodes per child-year, 95% CI 0·12–0·19) and in young infants (<6 months; 0·15 episodes per child-year, 0·12–0·19; table 2). RSV LRTI had a more severe clinical presentation and wheezing, requiring supplemental oxygen and hospitalisation compared with non-RSV LRTI (appendix p 2). Most children had a single episode of RSV LRTI (140 [92%] of 152); 12 (8%) children had second RSV LRTI events.

Recurrent LRTI occurred in 209 (18%) of 1131 children, with 116 (10%) having two and 93 (8%) children having three or more LRTIs. When categorising children according to their first LRTI with follow-up to 2 years, those with RSV were three times more likely to develop recurrent LRTI compared with those with non-RSV LRTI (0·32, 0·22–0·48 *vs* 0·10, 0·07–0·16, episodes per child-year; p<0·0001; appendix p 3). The incidence of recurrent LRTI was higher following all-cause hospitalisation than ambulatory LRTI (0·14, 0·09–0·21 *vs* 0·06, 0·04–0·09, episodes per child-year; p=0·003); this pattern was similar for RSV LRTI (appendix p 3). However, incidence of recurrent LRTI was much higher following hospitalised RSV LRTI (0·12 episodes per child-year, 0·06–0·24) compared with that following hospitalised non-RSV LRTI (0·02 episodes per child-year, 0·01–0·09; p=0·012; appendix p 3).

786 wheezing episodes occurred in 421 (37%) of 1131 children with a valid qPCR. Wheezing at LRTI occurred in 319 (41%) of 785 episodes, higher in those with RSV LRTI (98 [58%] of 164) compared with non-RSV LRTI (224 [36%] of 621; p<0·0001; IRR 1·40, 95% CI 1·24–1·57). Wheezing at the time of rhinovirus LRTI occurred in 182 (48%) of 377 children compared with 95 (60%) of 164 of those with RSV LRTI (p=0·014). Among hospitalised LRTI cases, wheezing occurred in 35 (65%) of 54 with RSV compared with 44 (40%) of 109 without RSV (IRR 1·29, 95% CI 1·09–1·52; p=0·0029). When comparing parental report with health worker ascertained wheeze at presentation to a health-care facility, there was high agreement between parentally reported and health-care worker ascertained wheezing (kappa 0·764).

**Figure 2:**
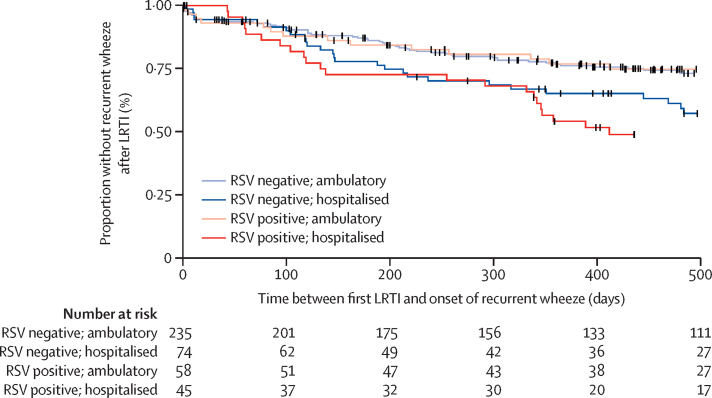
Kaplan-Meier estimates of time from first LRTI and onset of recurrent wheezing stratified by RSV association (at first LRTI episode) and severity (hospitalisation at first LRTI episode) Child follow-up period was divided into intervals of 2 weeks that were used to calculate person-time at risk, with a not at risk (excluded person-time) period of 28 days after a wheezing event. LRTI=lower respiratory tract infection. RSV=respiratory syncytial virus. DCHS=Drakenstein Child Health Study.

Over 40% (185 [44%]) of 420 children who experienced any wheezing had recurrent episodes. Overall, children who never had a LRTI (21 [3%] of 690) were less likely to develop recurrent wheezing compared with children who had non-RSV LRTI (76 [28%] of 274) or RSV LRTI (66 [43%] of 152), all pairwise; p<0·0001 (figure 1). More children experienced recurrent wheezing after hospitalised LRTI (62 [48%] of 130) compared with ambulatory LRTI (80 [25%] of 323; p<0·0001). This pattern of recurrent wheezing occurred in children hospitalised for RSV LRTI (29 [55%] of 53) compared with ambulatory RSV LRTI (37 [37%] of 99; p=0·040), in those hospitalised for non-RSV LRTI (21 [43%] of 49)versus ambulatory non-RSV LRTI (48 [21%] of 225; p=0·0020) and in those with rhinovirus LRTI (99 [39%] of 253). Time to recurrent wheezing after LRTI was more rapid and occurred in a higher proportion of children hospitalised with RSV LRTI compared with all other groups, with ambulatory non-RSV LRTI having the lowest likelihood of recurrent wheezing (figure 2, table 4). On multivariable analysis, risk factors associated with recurrent wheezing after LRTI included RSV (IRR 1·41, 95% CI 1·25–1·59) or hospitalisation (1·48, 1·30–1·68; table 4). These findings were similar when recurrent wheezing was defined as two or more or three or more episodes of wheezing and persisted after adjustment for a range of covariates (table 4).

**Table 4:** Multivariable models of the association between LRTI and subsequent risk of wheezing by categories of LRTI in the DCHS

	**Any wheezing**				**Recurrent wheezing (two or more events)**				**Recurrent wheezing (three or more events)**			
	Crude		Adjusted		Crude		Adjusted		Crude		Adjusted	
	IRR (95% CI)	p value	IRR (95% CI)	p value	IRR (95% CI)	p value	IRR (95% CI)	p value	IRR (95% CI)	p value	IRR (95% CI)	p value
**All LRTI**												
LRTI *vs* no LRTI	5·24 (4·37–6·28)	<0·0001	7·71 (6·35–9·35)	<0·0001	4·64 (3·59–6·00)	<0·0001	2·83 (2·31–3·47)	<0·0001	5·77 (3·82–8·70)	<0·0001	3·66 (2·61–5·15)	<0·0001
Hospitalised LRTI *vs* ambulatory LRTI	1·77 (1·45–2·17)	<0·0001	2·16 (1·75–2·65)	<0·0001	2·45 (2·04–2·95)	<0·0001	1·48 (1·30–1·68)	<0·0001	3·20 (2·28–4·47)	<0·0001	1·96 (1·52–2·53)	<0·0001
**RSV LRTI**												
RSV LRTI vs non-RSV LRTI	1·90 (1·58–2·29)	<0·0001	2·68 (2·19–3·37)	<0·0001	2·18 (1·89–2·50)	<0·0001	1·41 (1·25–1·59)	<0·0001	2·09 (1·66–2·63)	<0·0001	1·42 (1·16–1·75)	<0·0001
Hospitalised RSV LRTI *vs* non-RSV LRTI	1·69 (1·27–2·25)	<0·0001	2·72 (2·02–3·68)	<0·0001	2·03 (1·64–2·50)	<0·0001	1·32 (1·11–1·57)	0·0001	2·16 (1·54–3·02)	<0·0001	1·51 (1·12–2·05)	0·0080
Hospitalised RSV LRTI *vs* ambulatory RSV LRTI	1·73 (1·32–2·27)	<0·0001	2·04 (1·53–2·73)	<0·0001	2·54 (1·98–3·26)	<0·0001	1·50 (1·25–1·80)	<0·0001	3·12 (1·99–4·89)	<0·0001	1·92 (1·36–2·70)	<0·0001
**Rhinovirus LRTI**												
rhinovirus LRTI vs non-rhinovirus LRTI	2·07 (1·74–2·46)	<0·0001	3·39 (2·81–4·09)	<0·0001	2·39 (2·08–2·77)	<0·0001	1·65 (1·46–1·86)	<0·0001	2·49 (1·97–3·14)	<0·0001	1·82 (1·50–2·20)	<0·0001
Hospitalised rhinovirus LRTI *vs* non-rhinovirus LRTI	2·29 (1·81–2·90)	<0·0001	3·24 (2·53–4·16)	<0·0001	2·17 (1·83–2·58)	<0·0001	1·45 (1·26–1·68)	0·0001	2·48 (1·78–3·45)	<0·0001	1·68 (1·27–2·23)	<0·0001
Hospitalised rhinovirus LRTI *vs* ambulatory rhinovirus LRTI	2·13 (1·65–2·75)	<0·0001	2·59 (2·00–3·67)	<0·0001	2·65 (2·11–3·33)	<0·0001	1·61 (1·37–1·90)	0·0001	3·07 (2·09–4·52)	<0·0001	1·94 (1·41–2·66)	<0·0001
**RSV LRTI *vs* rhinovirus LRTI**												
RSV LRTI *vs* rhinovirus LRTI	0·89 (0·69–1·16)	0·39	0·92 (0·70–1·20)	0·52	0·91 (0·69–1·22)	0·54	1·11 (0·86–1·42)	0·43	0·86 (0·50–1·47)	0·58	1·12 (0·65–1·93)	0·68
Hospitalised RSV LRTI *vs* rhinovirus LRTI	0·94 (0·63–1·39)	0·76	0·76 (0·46–1·24)	0·27	0·83 (0·55–1·24)	0·36	1·66 (1·00–2·75)	0·051	0·65 (0·28–1·51)	0·32	0·42 (0·17–1·08)	0·071
Ambulatory RSV LRTI *vs* rhinovirus LRTI	1·05 (0·76–1·46)	0·78	1·12 (0·80–1·57)	0·51	1·23 (0·77–1·99)	0·39	1·08 (0·76–1·55)	0·66	1·48 (0·58–3·81)	0·41	1·74 (0·67–4·51)	0·26

Results of generalised estimating equations are incidence rate ratios (IRRs) and 95% CI. Models adjusted for sex, socioeconomic status, presence of young children in household, HIV exposure, maternal smoking, low birthweight, season of exposure, and age at time of wheezing episode or mid interval if no wheezing event. LRTI=lower respiratory tract infection. RSV=respiratory syncytial virus. DCHS=Drakenstein Child Health Study.

Lung function measurements were successfully obtained in more than 80% of children tested (815 [90%] at 6 weeks, 683 [87%] at 1 year, and 619 [83%] at 2 years). All-cause LRTI was associated with an average 3% higher respiratory rate (95% CI 0·01 to 0·06; p=0·013) and lower compliance (–0·1, −0·18 to −0·02) at 2 years compared with no LRTI, independent of baseline lung function (table 5). Recurrent episodes of LRTI were associated with further increased respiratory rate (0·01, 0·001 to 0·02) and respiratory system resistance (0·77 hPa s L^−1^, 0·07 to 1·47) and lower compliance (–0·6 mL h Pa^−1^, −0·09 to −0·02) with each additional event. No differences in lung function outcomes existed between children who had a RSV LRTI compared with those with a non-RSV LRTI (table 5).

**Table 5:** Comparison of lung function at 2 years of age by previous LRTI in the DCHS

	**LRTI *vs* no LRTI**		**Hospitalised LRTI *vs* ambulatory LRTI**		**RSV LRTI *vs* non-RSV LRTI**		**Change per additional LRTI episode**	
	Coefficient (95% CI)	p value	Coefficient (95% CI)	p value	Coefficient (95% CI)	p value	Coefficient (95% CI)	p value
Functional residual capacity	0·44 (−3·53 to 4·42)	0·83	1·70 (−5·80 to 9·20)	0·66	4·70 (−2·20 to 11·59)	0·18	−0·46 (−2·11 to 1·18)	0·58
Lung clearance index (n turnovers)	0·03 (−0·02 to 0·08)	0·23	0·06 (−0·03 to 0·15)	0·20	0·05 (−0·03 to 0·12)	0·25	0·02 (0·003 to 0·04)	0·026
Tidal volume (mL)	−0·69 (−2·08 to 0·69)	0·33	1·33 (−1·16 to 3·82)	0·30	0·09 (−2·39 to 2·22)	0·94	−0·51 (−1·08 to 0·07)	0·083
Log respiratory rate (breaths per min)	0·03 (0·01 to 0·06)	0·013	−0·02 (−0·07 to 0·02)	0·26	−0·02 (−0·06 to 0·02)	0·28	0·01 (0·001 to 0·02)	0·034
Log ratio time of total expiratory flow to time of expiration (%)	−0·01 (−0·05 to 0·02)	0·45	−0·04 (−0·11 to −0·03)	0·23	−0·03 (−0·09 to 0·03)	0·35	−0·01 (−0·03 to 0·002)	0·083
Fractional exhaled nitric oxide	−0·48 (−1·54 to 0·58)	0·38	0·14 (−1·71 to 1·99)	0·88	1·37 (−0·33 to 3·08)	0·12	−0·19 (−0·63 to 0·25)	0·39
Resistance (hPa sL^−1^)	1·42 (−0·23 to 3·07)	0·092	−0·45 (−3·53 to 2·63)	0·77	0·61 (−2·17 to 3·39)	0·67	0·77 (0·07 to 1·47)	0·030
Compliance (mL hPa^−1^)	−0·10 (−0·18 to −0·02)	0·014	0·01 (−0·14 to 0·15)	0·94	0·01 (−0·12 to 0·14)	0·92	−0·06 (−0·09 to −0·02)	<0·0001

Analyses used mixed-effects linear regression modelling with random subject effects, results presented as coefficients and 95% CI. Lung function measures included were collected at 6 weeks, 1 year, and 2 years. Models adjusted for body-mass index for age *Z* score, ethnicity, sex, socioeconomic quartile, age at LF visit and gestational age at birth. LRTI=lower respiratory tract infection. DCHS=Drakenstein Child Health Study. RSV=respiratory syncytial virus. LF=lung function.

## Discussion

This birth cohort study found that RSV is a major cause of LRTI in young children in South Africa, occurring in approximately 20% of episodes. Despite almost no HIV infection in children, good nutrition, and excellent immunisation coverage including Hib and PCV13, LRTI was very common. Although the incidence and severity of RSV LRTI was highest in infants under 6 months, RSV continued to be associated with approximately a fifth of LRTI up to age 2 years. Importantly, a first episode of RSV LRTI was associated with a substantially increased risk of subsequent non-RSV LRTI, especially if the first LRTI required hospitalisation. RSV was also associated with recurrent wheezing in early childhood, with the highest proportion of recurrent wheezing following RSV LRTI hospitalisation. However, rhinovirus LRTI or hospitalisation with non-RSV LRTI was also associated with an increased risk of recurrent wheezing. LRTI, particularly recurrent illness was also associated with impairments in lung function at 2 years, but this was not specific to RSV. Taken together, these data shed new light on the epidemiology of RSV LRTI and association with subsequent recurrent LRTI, wheezing, and lung function impairment.

Our data underscore the importance of RSV-associated LRTI in the context of high immunisation coverage for Hib and PCV13.^1^ In the PERCH study,^2^ RSV was the dominant pathogen, being attributed to 31% of severe or very severe hospitalised pneumonia episodes across seven LMICs, similar to our finding of 25% RSV prevalence in hospitalised cases. The finding that RSV causes a substantial proportion of LRTI through early childhood has important implications for novel preventive interventions under development. Maternal immunisation against RSV during late pregnancy or use of long-acting monoclonal antibodies shortly after birth might be effective to prevent disease in young infants.^19^ However, additional strategies such as follow-on immunisation of infants will be needed to prevent the burden occurring later in childhood.^20^ The impact of preventive strategies on the epidemiology of RSV LRTI and the importance of late onset RSV LRTI on recurrent wheezing requires further study.^20^

Despite the severity of disease associated with RSV LRTI in infants, mortality was low, reflecting timely access to appropriate therapy, careful follow-up of children, and low prevalence of risk factors such as HIV infection or severe malnutrition. However, RSV LRTI was associated with subsequent morbidity including recurrent LRTI and recurrent wheezing. Our results are similar to those of a cohort study of Gambian children hospitalised with RSV LRTI and age-matched controls, which reported an increased incidence of LRTI following hospitalised RSV LRTI in young children.^21^ The association of RSV LRTI with recurrent non-RSV LRTI might have important consequences, and is consistent with data in the Gambian study,^21^ especially as LRTI and particularly recurrent disease were associated with lung function impairments at 2 years. Novel preventive strategies for RSV under development might therefore potentially have a bigger impact on lung health than solely prevention of RSV LRTI.

Children hospitalised with RSV LRTI also had a higher prevalence of recurrent wheezing than those with ambulatory RSV LRTI, consistent with several studies reporting an association between hospitalisation for RSV and subsequent recurrent wheezing.^6^, ^22^ However, this pattern also occurred in rhinovirus-associated LRTI and other non-RSV LRTI, suggesting that the severity of LRTI, as reflected by hospitalisation, is a key factor associated with recurrent wheezing in young children. A key unresolved question is whether the association of RSV LRTI with recurrent wheezing reflects an underlying genetic or physiological susceptibility to wheezing, with RSV being the initial manifestation of such susceptibility, or whether RSV is part of a causal pathway leading to recurrent wheezing.^4^, ^23^ The former possibility is supported by data from twin registry studies.^4^, ^24^, ^25^ Several lines of evidence from our study suggest that the link between RSV and recurrent wheezing might not simply reflect underlying susceptibility. First, evidence for a genetic predisposition to recurrent wheezing, as assessed by maternal or family history of asthma or allergy, was uncommon, although this measurement of genetic susceptibility is limited. Second, children hospitalised with RSV LRTI had an increased risk of recurrent wheezing compared with children hospitalised with non-RSV LRTI, suggesting that RSV infection confers particular risk of recurrent wheezing. This increase also occurred following rhinovirus LRTI, consistent with data from high income countries in which the risk of recurrent wheezing is associated with early life RSV or rhinovirus LRTI.^26^, ^27^ Last, impairments in lung function that occurred following LRTI were independent of baseline lung function, suggesting that the risk of recurrent wheezing does not reflect underlying lung structural or functional impairment.

An alternative explanation is that RSV might lead to changes in immunological pathways or lung function that subsequently predispose to LRTI or recurrent wheezing.^4^, ^23^ However, although LRTI in early life was associated with impairments in lung function at 2 years, this association was a general effect of LRTI not of RSV. Importantly, recurrent LRTI, which was more common after an initial RSV LRTI, was associated with further reductions in lung function. These results are consistent with our previous findings that LRTI reduced lung function at 1 year of age and that recurrent LRTI impaired lung function further.^28^ RSV disease is likely to be both a marker of underlying susceptibility and a component of the causal pathway to recurrent wheezing or asthma.^4^

Factors underlying the development of recurrent wheezing may differ in LMICs compared to high-income countries. However, even in high-income countries, early-life LRTI has increasingly been associated with development of chronic adult respiratory disease.^29^ Our study provides further evidence that severe early-life LRTI is associated with recurrent wheezing and with reductions in lung function in early childhood.^30^ These data have important implications for understanding the determinants of chronic lung disease, suggesting that early-life LRTI leads to persistent impairments in lung function^29^, ^30^ and adding to the evidence for the early origins of asthma or chronic obstructive pulmonary disease. Further long-term study of these outcomes will be paramount to estimate the potential public health benefit of new RSV preventive interventions.

Limitations include a follow-up period of 2 years. Follow-up of this cohort with lung function will enable study of longer-term outcomes. A family history of asthma or atopy, which had a very low prevalence, was used as a proxy measure for genetic predisposition to wheezing. This measure is imperfect; we are planning genotyping and measurements of atopic status for future study. These limitations are mitigated by several strengths of this study. First, longitudinal study of LRTI and RSV LRTI allowed careful delineation of the epidemiology and characteristics of illness, and subsequent wheezing. Second, careful follow-up, with high cohort retention and active ascertainment of LRTI episodes and wheezing illness, make these data unique, especially in LMICs. Third, comprehensive longitudinal measurements of lung function allowed investigation of the impact of LRTI while adjusting for baseline lung function. Last, we used several different measures of recurrent wheezing illness (maternal report and health-care worker ascertainment), including two or three, or more episodes, with similar results. These results might not be generalisable to LMICs with lower levels of immunisation coverage or where Hib vaccine or PCV are not available. However, the uptake of Hib vaccine and PCV globally has increased, so these results are likely to be widely applicable in LMICs.

In summary, this study provides relevant new information on the epidemiology of RSV LRTI and the association with recurrent LRTI, wheezing, and lung function impairment. The findings that LRTI requiring hospitalisation, especially RSV LRTI, is associated with recurrent LRTI or recurrent wheezing has important consequences for child health. The impact of all cause early-life LRTI and recurrent LRTI on lung function provides further evidence of the importance of strengthening strategies to prevent LRTI and severe disease in young children. As survival of children improves, so the prevention of long-term morbidity from early-life LRTI becomes increasingly important. With several new RSV preventive strategies under development, the possibility that these approaches might be effective for primary prevention of acute RSV LRTI and for recurrent LRTI or wheezing requires further study.
