# Correction to *Lancet Glob Health* 2020; 8: e1316–25

**DOI:** 10.1016/S2214-109X(22)00088-2

**Published:** 2022-02-28

*Zar HJ, Nduru P, Stadler JAM, et al. Early-life respiratory syncytial virus lower respiratory tract infection in a South African birth cohort: epidemiology and effect on lung health.* Lancet Glob Health *2020; **8:** e1316–25*—The incidence of recurrent LRTI following hospitalised RSV LRTI compared with that following hospitalised non-RSV LRTI reported on page e1321 and in the appendix (p 3) of this Article has been corrected as of Feb 28, 2022.

