# Supplementary material for: Early-life respiratory syncytial virus lower respiratory tract infection in a South African birth cohort: epidemiology and effect on lung health
Source: Lancet Glob Health. 2020 Sep 21;8(10):e1316–25. doi: 10.1016/S2214-109X(20)30251-5 (PMC7511798; doi:10.1016/S2214-109X(20)30251-5)
Supplement: Supplementary appendix [file mmc1.pdf]

THE LANCET
Global Health

Supplementary appendix

This appendix formed part of the original submission and has been peer reviewed. We post it as supplied by the authors.

Supplement to: Zar HJ, Nduru P, Stadler JAM, et al. Early-life respiratory syncytial virus lower respiratory tract infection in a South African birth cohort: epidemiology and effect on lung health. *Lancet Glob Health* 2020; **8:** e1316–25.

Supplement Table 1: Results of unadjusted modelling of risk factors associated with any LRTI (Model A) and RSV-associated LRTI (Model B, C) in the first two years of life in the DCHS.

**Risk factor**	**Model A: LRTI vs no LRTI**		**Model B: RSV-LRTI vs non-RSV LRTI**		**Model C: RSV-LRTI vs no LRTI**	
	**Crude IRR (95% CI)**	**p-value**	**Crude IRR (95% CI)**	**p-value**	**Crude IRR (95% CI)**	**p-value**
Sex: Male (ref: female)	1·53 (1·26-1·85)	**<0·0001**	0·79 (0·64-0·98)	**0·033**	1·17 (0·88-1·58)	0·31
Season of birth (ref: Summer):						
Autumn (Mar-May)	1·39 (1·09-1·79)	**0·0090**	1·10 (0·85-1·43)	0·46	1·48 (1·03-2·11)	**0·032**
Winter (June-Aug)	0·93 (0·72-1·21)	0·61	0·83 (0·61-1·12)	0·22	0·67 (0·43-1·05)	0·22
Spring (Sept-Nov)	1·07 (0·81-1·40)	0·63	0·59 (0·38-0·90)	**0·015**	0·47 (0·27-0·81)	**0·0067**
Socio-economic quartile (ref: high):						
Lowest	1·40 (1·06-1·85)	**0·019**	0·77 (0·55-1·09)	0·14	1·20 (0·75-1·91)	0·46
Low- moderate	1·51 (1·15-1·99)	**0·0032**	0·92 (0·69-1·23)	0·58	1·49 (0·97-2·27)	0·068
Moderate- high	1·06 (0·80-1·40)	0·70	1·08 (0·81-1·45)	0·61	1·35 (0·87-2·09)	0·18
HIV Exposed (ref: HIV unexposed)	1·50 (1·22-1·83)	**0·0001**	0·81 (0·63-1·04)	0·097	1·10 (0·78-1·55)	0·57
Maternal Smoking in pregnancy (Self report): Smoker (ref: non-smoker)	1·04 (0·85-1·27)	0·82	1·31 (1·05-1·62)	**0·016**	1·41 (1·04-1·92)	0·029
Maternal Smoking post birth (Self report): Smoker (ref: non-smoker)	1·04 (0·85-1·27)	0·71	1·33 (1·07-1·65)	**0·0090**	1·33 (0·98-1·80)	0·063
Presence of other children in the household	1·04 (0·77-1·40)	0·79	1·12 (0·81-1·54)	0·49	1·72 (1·03-2·88)	**0·039**
Lower birth weight (<2·5kg)	1·63 (1·28-2·06)	**<0·0001**	0·83 (0·63-1·11)	0·21	1·26 (0·85-1·88)	0·25
Exclusive breastfeeding at 6 weeks (ref: no)	0·89 (0·74-1·08)	0·25	1·02 (0·82-1·27)	0·85	0·94 (0·73-1·21)	0·25
Exclusive breastfeeding at 3 months (ref: no)	1·04 (0·84-1·30)	0·70	0·95 (0·74-1·22)	0·71	1·02 (0·77-1·35)	0·91
Infant age (unit increase in years)	0·46 (0·40-0·52)	**<0·0001**	0·78 (0·64-0·96)	**0·020**	0·59 (0·53-0·65)	**<0·0001**
Preterm (<37 weeks' gestation)	1·51 (1·19-1·92)	**<0·001**	0·88 (0·67-1·14)	0·32	1·14 (0·78-1·68)	0·49

Table notes: Analyses done using generalized estimating equations; results presented as incidence rate ratios with 95% confidence intervals. Abbreviations: LRTI=lower respiratory tract infection; RSV=respiratory syncytial virus; HIV=human immunodeficiency virus; DCHS=Drakenstein Child Health Study

Supplement Table 2: Clinical characteristics of RSV-LRTI compared to non-RSV LRTI cases in the DCHS

	RSV LRTI n=164	Non-RSV LRTI n=621	P-Values
Median age at onset (IQR) days	198 (92 – 371)	213 (112 – 437)	0·074
Age category at presentation:			
0-1 years	121 (74)	408 (66)	0·050
1-2 years	43 (26)	213 (34)	
Site of recruitment:			
Mbekweni	90 (55)	394 (63)	**0·036**
TC Newman	74 (45)	227 (37)	
Season of presentation: Autumn (Mar-May)	82 (50)	140 (23)	**<0·0001**
Winter (June-Aug)	79 (48)	177 (29)	
Spring (Sept-Nov)	3 (2)	196 (32)	
Summer (Dec-Feb)	0 (0)	108 (17)	
**Presenting symptoms**			
Median temperature (°C) (IQR)	37 (37 – 38)	37 (36 – 37)	0·11
Median heart rate (IQR)	152 (136 – 164)	142 (132 – 160)	**<0·0001**
Median respiratory rate (bpm) (IQR)	56 (50 – 62)	54 (48 – 60)	**0·0012**
Oxygen saturation (%) (IQR)	97 (93 – 98)	98 (95 – 99)	**<0·0001**
Lower chest indrawing present	130 (80)	406 (67)	**0·0040**
Any oxygen received	32 (20)	56 (9)	**<0·0001**
Wheezing at time of LRTI	95 (58)	224 (36)	**<0·0001**
Admitted to hospital	54 (33)	109 (18)	**<0·0001**
Median days of hospitalisation (IQR)	4 (2-6)	3 (2-5)	0·12

Abbreviations: LRTI=lower respiratory tract infection; RSV=respiratory syncytial virus; IQR=interquartile range; bpm=beats per minute; DCHS=Drakenstein Child Health Study

Supplement Table 3: Incidence of recurrent LRTI following first LRTI in the DCHS

Category of First LRTI	Incidence (e/cy; 95% CI) of 2 or more LRTI episodes after 1st LRTI	P-value
RSV-LRTI	0·32 (0·22-0·48)	**<0·0001**
Non-RSV LRTI	0·10 (0·07-0·16)	
Hospitalised LRTI	0·14 (0·09 - 0·21)	**0·0031**
Ambulatory LRTI	0·06 (0·04 - 0·09)	
RSV-hospitalised LRTI	0·12 (0·06 - 0·24)	**0·012**
RSV- ambulatory LRTI	0·02 (0·01 - 0·09)	
RSV-hospitalised LRTI	0·12 (0·06 - 0·24)	**0·012**
Non-RSV hospitalised LRTI	0·02 (0·01 - 0·09)	

Abbreviations: LRTI=lower respiratory tract infection; e/cy = episodes per child-year; CI=confidence interval; RSV=respiratory syncytial virus; DCHS=Drakenstein Child Health Study
